# Immediate Versus Semi-Elective Treatment of Stable Slipped Capital Femoral Epiphyses (SCFE)

**DOI:** 10.3390/children12070923

**Published:** 2025-07-11

**Authors:** Andrew G. Dubina, Alexandra M. Dunham, Julia L. Conroy, Karli M. Funk, Julio J. Jauregui, Paul D. Sponseller, Joshua M. Abzug

**Affiliations:** 1 Department of Orthopaedics, University of Maryland School of Medicine, Baltimore, MD 21201, USA; andrew_dubina@urmc.rochester.edu (A.G.D.); adunham@som.umaryland.edu (A.M.D.); jconroy@som.umaryland.edu (J.L.C.); karlifunk@som.umaryland.edu (K.M.F.); jabzug@som.umaryland.edu (J.M.A.); 2 Department of Orthopaedics, Johns Hopkins Children’s Center, Baltimore, MD 21287, USA; psponse@jhmi.edu

**Keywords:** immediate, SCFE, semi-elective, slipped capital femoral epiphysis, stable

## Abstract

**Background/Objectives**: Timing of fixation of stable slipped capital femoral epiphysis (SCFE) is controversial. As pressure mounts to limit inpatient admissions and procedures, our aim was to investigate whether treatment of SCFE in a delayed manner is a safe alternative to immediate fixation. Our hypothesis was that there would be no difference in complications for stable slips treated immediately (<24 h) versus semi-electively (>24 h) with screw fixation. **Methods**: A retrospective review was performed at two academic institutions during a 10-year-period yielding 91 SCFEs. Data collected included patient demographics, time to treatment, radiographic measurements (Southwick angle), and complications. Between-group analysis was performed using Welch’s *t*-test and Fisher’s exact test. **Results**: 91 stable SCFEs were identified with a median age of 12.3 years (IQR: 11.4–13.3). A total of 62 (68%) slips were treated immediately while 29 (32%) were treated in a semi-elective manner with a median time from diagnosis to surgery of 4 days (range: 2–11 days). There were no instances of >18° increase in Southwick angle in either group or conversion from stable to unstable slips during the semi-elective period. Overall, 12 (13%) patients experienced complications, but no difference in complication rate was observed between groups (15% vs. 10%, *p* = 0.75). However, the complication profile varied between groups. Of note, two patients (2%, 2/91) experienced AVN, both of which were treated in a semi-elective manner and underwent in situ pinning. **Conclusions**: There was no difference in complication rate between stable SCFEs treated immediately or semi-electively; however, the complication profile differed by group. No SCFEs in either group had >18° worsening of the Southwick angle between the time of diagnosis and the time of fixation and there were no conversions of stables slips to unstable slips while waiting for semi-elective surgery. These findings suggest that performing semi-elective surgical fixation within 11 days of diagnosing stable, mild SCFEs appears to be a safe alternative to inpatient admission at the time of diagnosis.

## 1. Introduction

Slipped capital femoral epiphysis (SCFE) is a developmental condition affecting primarily pubertal children, with a prevalence of 10.8 cases per 100,000 children [1]. SCFE is generally more common in males than females [1]. The incidence of SCFE has increased between 2011 and 2020 and has been associated with factors such as premorbid obesity and socioeconomic deprivation [2,3]. Slipped capital femoral epiphyses are commonly clinically defined by the Loder classification as being either “stable” (able to bear weight on the affected limb with or without crutches) or “unstable” (unable to bear weight), with a significant difference in the risk of osteonecrosis between groups (<1% vs. up to 47%, respectively) [4,5].

The standard of care for the management of SCFE is surgical stabilization, typically with screw fixation; however, the timing and method of surgical fixation is variable based on slip stability and the amount of slip deformity. Stable slips are commonly treated percutaneously with a single cannulated screw [6]. The timing of fixation for a stable slip is controversial. Immediate treatment (<24 h) would typically require presentation and admission through emergency services with inpatient surgical fixation and a variable length of post-operative inpatient recovery. Semi-elective treatment (>24 h) could include scheduled outpatient surgery, potentially at an ambulatory surgical center. A survey of the Pediatric Orthopedic Society of North America (POSNA) membership revealed that 81% of the respondents (*n* = 220) would treat a stable SCFE in an elective fashion, compared to only 5% of respondents that would treat unstable slips electively [7]. There is an increased interest in treating severe stable and unstable slips with an open reduction through a surgical hip dislocation and modified Dunn osteotomy as described by Ganz et al. [8]. Though severe slips may be treated with a modified Dunn osteotomy with success, complications such as avascular necrosis (AVN) and nonunion have been noted [6,9,10]. Of the participants in the POSNA survey, 100% would treat mild-to-moderate and 82% (*n* = 165/202 respondents) would treat severe stable SCFE with in situ fixation with or without manipulation [7].

As hospitalization costs increase and administrative pressure mounts to limit inpatient admissions and procedures [11,12,13], as well as patient/parent preferences to minimize costs and decrease hospitalization, the purpose of this study was to investigate whether treatment of SCFE in a semi-elective manner is a safe alternative to immediate treatment. The hypothesis developed was that there would be no difference in complications for stable slipped capital femoral epiphyses treated with screw fixation in an “immediate” (<24 h) or a “semi-elective” (>24 h) manner.

## 2. Materials and Methods

After institutional review board approval was obtained, a retrospective review of the billing database was performed at two academic institutions over a 10-year period (2011 to 2020) yielding 128 patients and 150 slipped capital femoral epiphyses, which were subsequently given patient IDs. Patients were excluded if their pre- or post-operative radiographs were unavailable for review (*n* = 32) or if they had less than 30 days of follow up (*n* = 9). Furthermore, 18 slips were characterized as “unstable” due to an inability to ambulate and were excluded from this investigation [4,5]. Therefore, the final study group comprised 91 stable slipped capital femoral epiphyses with an average follow up duration of 22 months (SD 26 months).

The 29 semi-elective SCFE surgeries occurred at an average of 5 days after the initial diagnosis was made (range 2–11 days). A total of 27 of the 29 semi-elective SCFE surgeries were treated as a scheduled outpatient surgery at the next available time. A total of 2 of the 29 semi-electively treated SCFE surgeries were admitted to the hospital through emergency services: one had delayed fixation despite emergent admission; the other was admitted after having been seen in an outpatient clinical setting two days prior to hospital admission.

Patient medical records were accessed for data collection, which included patient factors (age, gender, race, body mass index (BMI)), surgical factors (time of diagnosis and time of fixation), and follow up data including symptoms of impingement and pain. At the time of initial diagnosis, clinical documentation confirmed that all patients were instructed to be either toe-touch or non-weight bearing on crutches until the time of surgical fixation.

Radiographs, including those at the time of diagnosis and those at the time of fixation, were reviewed by a single observer at each study site (AGD and AMD, both PYG-4) and initially measured several examples together prior to quantifying the entire study population to minimize interobserver variability between study sites. The measurements were reviewed by attending physicians (PDS and JMA). The femoral diaphyseal/epiphyseal slip angle, as defined by Southwick [14], was measured for each SCFE on the lateral hip radiograph and additionally quantified as “mild” (<30°), “moderate” (30–50°), or “severe” (>50°). If plain radiographs or fluoroscopy were not taken immediately pre-operatively with the patient on the operating table, final radiographs taken after the fixation of the SCFE were used as a surrogate for the slip angle at the time of fixation.

Complications including femoral head chondrolysis, AVN, impingement, and/or other complications requiring return to the operating room (hardware prominence, fracture, etc.), were recorded as they were documented in the medical record at the clinic follow-up.

### Statistical Analysis

Simple statistical analysis was performed to determine means, standard deviations, and ranges for normally distributed continuous variables and counts and proportions for categorical variables. The Shapiro–Wilk test was utilized to assess the normality of continuous variables. Medians and interquartile ranges were calculated and reported for continuous variables that were not normally disturbed, including age, body mass index (BMI), and the duration of symptoms prior to diagnosis. A logistic regression model was utilized with patient age, sex, and BMI as covariates to test for confounders in our results. Between-group analysis was performed using Welch’s t-test for continuous variables with normal distributions, the Mann–Whitney U test for continuous variables that were not normally distributed, and Fisher’s exact test for categorical variables. These methods were utilized because equal standard deviation is not required between groups. The significance level was set at *p* < 0.05.

## 3. Results

A total of 91 stable slipped capital femoral epiphyses were included in the overall study cohort, of which 62% were male with a median age of 12.3 years (IQR: 11.4–13.3) (Table 1). Of the SCFEs investigated, 62 (68%) underwent immediate treatment, while 29 (32%) were treated semi-electively. All but one stable SCFE (99%) underwent in situ pinning. A total of 93% (*n* = 85) were fixed with a single 7.3 mm cannulated screw. Of the SCFEs treated immediately, 63% (*n* = 39/62) were graded as mild, 23% (*n* = 14) moderate, and 14% (*n* = 9) severe, compared to 93% (*n* = 27/29) considered mild and 7% (*n* = 2) severe in those treated semi-electively (Table 2).

Although there was a greater proportion of moderate and severe slips among the immediate treatment group, no statistically significant difference between the initial slip angle was observed between groups (30° vs. 25°, *p* = 0.19) (Table 2). There is also no significant difference in complication rate when taking possible confounding variables into account (Table 3). Those SCFEs that underwent immediate surgical management had a statistically significant greater correction of the Southwick angle at the time of surgery compared to those treated semi-electively (12° vs. 5°, *p* = 0.02). No SCFE in either group had greater than 18° of worsening Southwick angle between the time of diagnosis and the time of fixation, and there were no conversions of stable to unstable slips while waiting for semi-elective surgery.

Overall, there were 12 complications in the study (13% of patients) including 9 patients who required revision surgery. There was no statistically significant difference in complication rates between the patients that underwent immediate fixation compared to those treated in a semi-elective manner (15% vs. 10%; *p* = 0.75). There were no instances of the same complications reported between the immediate and semi-elective groups (Table 4). The semi-elective group had two (7%) cases of femoral head AVN and one (3%) instance of chondrolysis, which was treated non-operatively in a morbidly obese individual.

The overall rate of AVN in the study population was 2% (*n* = 2/91 patients). Of the two patients with clinical and radiographic AVN, both were 11 years old and underwent in situ pinning with a single 7.3 mm screw. Both patients (*n* = 2) were followed up with for at least 3 years after initial diagnosis. One child presented with an initial slip angle of 5° and was pinned at 4 days after diagnosis, ultimately requiring a Chiari salvage osteotomy; the other child had an initial slip angle of 53° and was pinned at 6 days, subsequently having a core decompression and bone marrow aspirate injection 20 months after in situ pinning.

## 4. Discussion

In the present study comparing stable SCFE treated either immediately (within 24 h after diagnosis) or semi-electively (>24 h), there was no difference in the complication rate (15% vs. 10%, *p* = 0.75) based on the timing of treatment. The complication profile did vary between groups, as those with early fixation tended to have implant-related complications compared to the semi-elective group, which either had AVN or chondrolysis. There was a higher proportion of moderate and severe SCFEs among the immediate group; however, there was no statistically significant difference in mean initial Southwick angle between groups. Patients treated immediately did have an improvement in the Southwick angle by a mean of 7° compared to the semi-elective group. No instances of >18° worsening of the Southwick angle or conversions from stable to unstable slips were reported throughout the semi-elective period prior to fixation.

Avascular necrosis of the femoral head, while more commonly observed after fixation of unstable slips (21% to 47%) is also observed following the fixation of stable SCFEs with a reported rate of 5.1% [4,15,16]. With respect to the treatment of stable SCFEs, AVN is likely related to direct injury to the vasculature at initial injury, injury related intracapsular hematoma with resultant pressure limiting vascular flow, or damage due to aberrant screw trajectory. Timing of surgical fixation of unstable SCFEs have been previously examined, with findings demonstrating greater rates of AVN when surgical fixation was performed between 24 h and 7 days, in contrast to surgical fixation performed either within 24 h or after 7 days of onset [17,18]. The “unsafe window” between 24 h and 7 days is hypothesized to be the result of elevated intra-articular pressure, which may elevate the potential risk for AVN [17]. However, there is a paucity of the literature investigating timing of stable SCFE fixation and rates of AVN, likely due to the rarer nature of this complication within this subgroup. Further investigation may be warranted due to discrepancies in rates of AVN elucidated between the immediate and semi-elective groups. The 7% rate of AVN among the semi-elective group is still comparable to the rate of 5.1% of stable SCFEs developing AVN reported in the literature [16].

The average Southwick slip angles for the immediate and semi-elective groups in the present study were 30°and 25°, respectively, and therefore classified as either a mild or moderate slip. There was a higher percentage of mild slips within the semi-elective group versus the immediate fixation group (93% vs. 63% mild slips). The previous literature reports that delayed the diagnosis of a SCFE have been associated with advancement of the Southwick angle, which was observed among patients that were treated in a semi-elective manner (average duration between diagnosis and surgery was 5 days, ranging 2–11 days) [19]. However, within this subgroup who experienced a worsening Southwick angle, none worsened more than 18° and no patients were converted from a stable to unstable classification. Perhaps some mild difference in angle measurement can be attributed to radiographic technique and rotation bias for clinic-obtained imaging versus postoperative radiographs taken in the operating room. Fedorak et al. described an assessment of 147 hips with a mean time of 20.9 days from diagnosis to surgery, finding that the worsening of the Southwick angle was associated with a further need for significant future surgery (osteotomy, arthroplasty, arthrodesis) [19]. At this time, further studies aimed at assessing the incidence of conversion from a stable to an unstable SCFE are warranted.

The correction of the slip angle between pre- and intra-operative imaging was significantly greater in those SCFE treated immediately compared to semi-electively (12° vs. 5°; *p* = 0.02). This difference in improvement can partially be explained by the treatment preferences between study centers, as the site that contributed the majority of patients in the “immediate” fixation group tended to do the pinning on the fracture table compared to supine on a flat table at the study site that contributed more patients to the “semi-elective” group. Furthermore, the goal in each of these cases was in situ fixation with possible gentle manipulation with positioning of the leg; however, no attempt at acute open surgical correction was made. Given there is no published minimally clinically important difference (MCID) or mean detectable change (MDC) for the Southwick diaphyseal/epiphyseal slip angle, it is unclear whether the 7° difference in improvement in slip angle between groups represents a clinically important difference or whether or not the 18° difference in slip angle within the semi-elective group from time to diagnosis until surgery represents a clinically important difference.

As costs of hospitalization continue to rise, the transition of traditional inpatient procedures to outpatient surgeries may result in a reduction of overall cost. Though there is limited data regarding the cost and length of stay for outpatient in situ fixation for SCFE, a recent study conducted in Finland determined the mean length of stay to be 3.4 days after in situ fixation [20]. While the literature does not currently demonstrate a comparison between inpatient and outpatient costs of in situ fixation for SCFE, cost analyses of inpatient vs. outpatient joint arthroplasties have depicted a 20–30% decrease in overall cost when surgeries were performed in the outpatient setting [21]. Further, a systematic review conducted by Crawford et al. found cost savings of up to 60% with outpatient procedures and similar or improved patient satisfaction [22]. These findings suggest that surgical fixation of stable SCFE in the outpatient setting may yield considerable reduction in cost.

Limitations of the current study include its retrospective nature and all biases inherent to this study design. The study is not randomized, therefore each of the treatment centers involved in the study had a bias towards their preferred timing of treatment. The slip angle measurements were completed on the available imaging by comparing the pre-operative and intra-operative images. If no intra-operative fluoroscopy images were available, it was assumed that no significant reduction was performed at the time of fixation since these were stable, mild SCFES; in those cases, post-operative images were used. Given that there were no open reductions performed in this study group, the incidental reduction based on patient positioning would be unlikely to significantly change the slip angle and therefore, the immediate post-operative slip angle can be an acceptable surrogate to that which is present at the time of surgery. Additionally, the study has a limited number of patients over a 10-year period and therefore additional studies are required to confirm these findings. Future studies with a larger number of patients may find additional significant differences in complication rates between the timing of treatment groups or confounding variables.

## 5. Conclusions

In this study’s population of 91 stable slipped capital femoral epiphyses, no significant difference was observed between complication rates of the immediate and semi-elective treatment groups; however, there was a difference in complication profile. There was a greater proportion of moderate and severe slips among the immediate treatment group; however, there was no significant difference in mean initial Southwick angle. No instances of >18° worsening of the Southwick angle or conversions from stable to unstable slips were reported in the semi-elective treatment group during the duration prior to surgery. Future larger or randomized prospective studies may provide further information surrounding the risks associated with the timing of fixation of SCFEs. Based on the results of this data, semi-elective surgical fixation of a stable, mild SCFE appears to be a safe and likely cost-effective alternative to inpatient admission at the time of diagnosis.

## Figures and Tables

**Table 1:** Patient characteristics.

Median (IQR)/*n* (%)	Overall Stable Slips (*n* = 91)	Immediate (*n* = 62) (<24 h)	Semi-Elective (*n* = 29) (>24 h)	*p*-Value
Age (year)	12.3 (11.4–13.3)	11.4 (11.4–12.5)	12.8 (11.8–13.5)	0.53
Gender	56 (62%) Male 35 (38%) Female	36 (58%) Male 26 (42%) Female	20 (69%) Male 9 (31%) Female	0.46
Side of slip	43 (47%) Right 48 (53%) Left	26 (42%) Right 36 (58%) Left	17 (58%) Right 12 (42%) Left	0.28
BMI	35.1 (28.2–39.3)	29.8 (28.3–36.3)	35.8 (28.0–42.8)	0.64
Duration of symptoms prior to diagnosis (months)	2.0 (1.0–3.5)	2.0 (2.0–3.0)	1.5 (0.9–3.5)	0.37

BMI = body mass index.

**Table 2:** Comparison of slip characteristics between immediate (<24 h) and semi-elective (>24 h) treatment groups.

Average (SD)/N (%)	Immediate (*n* = 62)	Semi-Elective (*n* = 29)	*p*-Value
Initial slip angle (°)	30 (18)	25 (16)	0.19
Slip angle at time of surgery (°)	17 (16)	19 (17)	0.60
Correction of slip angle (initial-at time of surgery) (°)	12 (13)	5 (13)	0.02
Severity of slip angle			<0.01
Mild	39 (63%)	27 (93%)	
Moderate	14 (23%)	0 (0%)	
Severe	9 (14%)	2 (7%)	

° = degrees.

**Table 3:** Logistic regression test.

Covariant	Regression Coefficient	Standard Error	*p*-Value	Odds Ratio	97.5% CI Lower	97.5% CI Upper
Constant	−5.7254	4.364	0.190		−14.278	2.827
Age	0.3998	0.307	0.192	1.533433	−0.201	1.001
Gender	−1.5233	0.902	0.091	0.689741	−3.291	0.245
BMI	−0.0089	0.050	0.860	0.917736	−0.107	0.090

BMI: body mass index; CI: confidence interval.

**Table 4:** Postoperative complications.

Patient ID	Complication	Intervention	Group
32	Cam-type deformity	Arthroscopic debridement, osteoplasty	Immediate
79	Screw tip broken, progressive deformity	Femoral osteotomy	Immediate
88	Screw prominence	Observation	Immediate
6	Limb length inequality, external rotation deformity	Southwick osteotomy	Immediate
58	Mild cam-type deformity	Observation	Immediate
60	Screw prominence	ROH	Immediate
69	Periprosthetic subtrochanteric femur fracture (1-month post-op)	Open reduction internal fixation	Immediate
75	Cam-type deformity	Arthroscopic debridement, osteoplasty	Immediate
125	FAI	Proximal femoral osteotomy, femoral head reshaping	Immediate
7	Chondrolysis	Observation Morbidly obese	Semi-elective
65	AVN, saddle deformity	ROH, Chiari osteotomy	Semi-elective
115	AVN	Osteochondroplasty, core decompression with iliac crest autograft, revision 2 years later	Semi-elective

FAI = femoroacetabular impingement; AVN = avascular necrosis; ROH = removal of hardware.

## Data Availability

All data and materials support their published claims and comply with field standards and are available for review.
